# Evaluation of the Lethal Potency of Scorpion and Snake Venoms and Comparison between Intraperitoneal and Intravenous Injection Routes

**DOI:** 10.3390/toxins6061873

**Published:** 2014-06-12

**Authors:** Naoual Oukkache, Rachid El Jaoudi, Noreddine Ghalim, Fatima Chgoury, Balkiss Bouhaouala, Naima El Mdaghri, Jean-Marc Sabatier

**Affiliations:** 1 Laboratory of Venoms and Toxins, Pasteur Institute of Morocco, 1 Place Louis Pasteur, Casablanca 20360, Morocco; E-Mails: nghalim@yahoo.fr (N.G.); fchgoury@gmail.com (F.C.); naima.elmdaghri@gmail.com (N.E.M.); 2 Laboratory of Pharmacology and Toxicology, Faculty of Medicine and Pharmacy, University Mohamed V-Souissi, Rabat 6203, Morocco; E-Mail: eljaoudi_rachid@yahoo.fr; 3 Laboratory of Venoms and Therapeutic Molecules, Pasteur Institute of Tunis, University of Tunis El Manar, 13 Place Pasteur, BP74, Tunis 1002, Tunisia; E-Mail: balkiss.bouhaouala@gmail.com; 4 Laboratory INSERM UMR 1097, University of Aix-Marseille, 163, Parc Scientifique et Technologique de Luminy, Avenue de Luminy, Bâtiment TPR2, Case 939, Marseille 13288, France; E-Mail: sabatier.jm1@libertysurf.fr

**Keywords:** scorpion, snake, venoms, animal toxins, lethal activity, toxicity

## Abstract

Scorpion stings and snake bites are major health hazards that lead to suffering of victims and high mortality. Thousands of injuries associated with such stings and bites of venomous animals occur every year worldwide. In North Africa, more than 100,000 scorpion stings and snake bites are reported annually. An appropriate determination of the 50% lethal doses (LD_50_) of scorpion and snake venoms appears to be an important step to assess (and compare) venom toxic activity. Such LD_50_ values are also commonly used to evaluate the neutralizing capacity of specific anti-venom batches. In the present work, we determined experimentally the LD_50_ values of reference scorpion and snake venoms in Swiss mice, and evaluated the influence of two main venom injection routes (*i.e.*, intraperitoneal (IP) *versus* intravenous (IV)). The analysis of experimental LD_50_ values obtained with three collected scorpion venoms indicates that *Androctonus mauretanicus* (Am) is intrinsically more toxic than *Androctonus australis hector* (Aah) species, whereas the latter is more toxic than *Buthus occitanus* (Bo). Similar analysis of three representative snake venoms of the Viperidae family shows that *Cerastes cerastes* (Cc) is more toxic than either *Bitis arietans* (Ba) or *Macrovipera lebetina* (Ml) species. Interestingly, the venom of Elapidae cobra snake *Naja haje* (Nh) is far more toxic than viper venoms Cc, Ml and Ba, in agreement with the known severity of cobra-related envenomation. Also, our data showed that viper venoms are about three-times less toxic when injected IP as compared to IV, distinct from cobra venom Nh which exhibited a similar toxicity when injected IP or IV. Overall, this study clearly highlights the usefulness of procedure standardization, especially regarding the administration route, for evaluating the relative toxicity of individual animal venoms. It also evidenced a marked difference in lethal activity between venoms of cobra and vipers, which, apart from the nature of toxins, might be attributed to the rich composition of high molecular weight enzymes in the case of viper venoms.

## 1. Introduction

Envenoming caused by venomous animals is occurring in numerous regions worldwide. These serious events, caused mainly by scorpions and snakes, are responsible for a high annual death rate [1,2]. A vast number of people are actually exposed to possible envenomation. In most cases, local population is not well protected against the envenoming risk, and envenomed patient often needs to benefit from urgent symptomatic and immunotherapeutic treatments.

In Maghreb countries (e.g., Mauritania, Morocco, Algeria, Tunisia and Libya), at least 100,000 victims of envenomation are reported annually, of which 150 died. The Central Africa (e.g., Ghana and Senegal), Middle East (e.g., Iran), Central and South America (e.g., Brazil, and Mexico) are also affected. Severity of envenoming is remarkable in MATI region (*i.e.*, Morocco, Algeria, Tunisia and Iran). However, epidemiologic data are often scarce due to under-declarations and limited studies [3,4].

Among scorpions living in North Africa, twelve species are very important and involved in severe accident cases: *Androctonus australis*, Aa; *Androctonus mauretanicus*, Am; *Androctonus crassicauda*, Ac; *Buthus occitanus*, Bo; *Odontobuthus doriae*, Od; *Hottentotta saulcyi*, Hs; *Hottentotta schach*, Hsch; *Hottentotta jayakari*, Hj; *Mesobuthus eupeus*, Me; *Hemiscorpius lepturus*, He; *Hemiscorpius persicus*, Hp; *Leiurus quinquestriatus*, Lq). Their venom toxicity is variable and also depends on the injection route and animal body weight [3,4].

There are over 400 species of front fanged snakes but only a few are known to be dangerous. The foremost medically important species in North Africa belong to the Viperidae family (e.g., *Cerastes cerastes*, Cc; *Bitis arietans*, Ba; *Vipera lebetina mauretanica* (*Macrovipera lebetina*, Ml) which is responsible for hemorrhagic effects, whereas the Elapidae family (e.g., *Naja haje*, Nh) is mostly known for its neurotoxic effects. The majority of snake bites occur in rural areas. They are inflicted on the feet or ankles and occurring most of the time during the evening hours when people tread on snakes, and sometimes at night while sleeping or moving [4,5].

Hence, fight against envenoming is a priority-public health issue in several countries [6,7]. As part of the combat, appropriate assessment of scorpion and snake venom 50% lethal doses (LD_50_) is an important step for an accurate evaluation of the toxic activity of specific venoms, and is also regularly used to select the relevant anti-venom batch, as well as to establish the neutralizing capacity of vials.

According to the World Health Organization, venom lethality is expressed as median lethal dose (LD_50_). The LD_50_ value is defined as the amount of a substance (or venom) causing death of 50% injected mice [4,7,8,9,10,11,12,13].

Venom median lethal dose (LD_50_) assessment is useful for the determination of the scorpion or snake venom toxicity, as well as potency of anti-venom serum. The animal venom “pool” is also used as an immunogenic mixture for anti-venom production.

Since, the LD_50_ value of an given animal venom is often determined and reported differently by investigators, we decided to more deeply investigate the influence of intraperitoneal *versus* intravenous administration route for calculating lethal potency of snake and scorpion venoms. Depending on the research teams (with more or less distant geographical locations), could be significantly different (i) the method used to milking scorpion and collecting venom; (ii) the mouse species and number of mice used for LD_50_ value determination; and (iii) the route of venom injection for testing toxicity.

Five distinct routes can be used for venom injection, *i.e.*, intracerebroventricular (ICV), intramuscular (IM), intravenous (IV), intraperitoneal (IP), and subcutaneous (SC). The lowest LD_50_ value is always obtained by the ICV route, whereas the SC route gives the highest LD_50_ value.

Here we studied the relative toxicities of scorpion (Am, Ah and Bo species) and snake (Nh, Ml and Cc species) venoms in mice, based on the determination of their LD_50_ values in two common routes of venom injection (IP and IV) [4,13].

## 2. Results and Discussion

The toxicity evaluation of scorpion or snake venom is a critical step for an efficient determination of the venom activity. Diverse methodologies and experimental protocols have been used to determine the median lethal dose (LD_50_ value) of the venom. Several animal models such as *Sarcophaga argyrostoma* larvae of the blowfly, *Musca domestica* fly larvae, adult *Blatella germanica* cockroach, chick, rat and guinea-pig, have been used. The most common model for venom toxicity analyses is the LD_50_ value determination in mice [2,8,9,14].

However, the individual, seasonal and geographical variability of venom, the type/species of the targeted animal and its body weight, the route of venom injection and the experimental strategies that followed venom recovery, are the main variables which potentially impact the LD_50_ values of venoms [7,15].

In this study, the LD_50_ mean values of venoms were assessed by both the intravenous and intra-peritoneal routes using 20 ± 2 g Swiss mice. Generally, the determination of venom lethality via IV injection allows knowing the actual toxic effects when bioavailability of venom components in the blood is complete [4,8,10].

### 2.1. Lethal Potency of Scorpion Venoms

The LD_50_ toxicity values of collected scorpion venoms were assessed by IP or IV routes in ca. 20 g Swiss mouse and summarized in Table 1. The ratios of LD_50_ values obtained via IP and IV injections in mice were calculated. According to the two injection routes, experimental data indicate that the most toxic is the Am venom, whereas the less toxic is Bo venom. The Am venom is over three-times more toxic than the Bo venom. Similarly, slightly less potent Aah venom is approximately three-times more toxic that the Bo venom.

**Table 1:** Median lethal doses (LD_50_) of Am, Bo and Aah scorpion venoms, as determined using IV and IP injection routes, with 95% confidence intervals and calculated by non-linear regression.

	LD_50_		
	Am Venom	Bo Venom	Aah Venom
IV (µg/mouse)	4.7 (4.1–5.4)	15.2 (14.8–15.6)	5.2 (4.7–5.7)
IV (µg/kg)	235 (205–270)	775 (740–780)	260 (235–285)
IP (µg/mouse)	5.8 (5.3–6.4)	17.1 (16.7–17.5)	6.7 (6.4–7.1)
IP (µg/kg)	290 (265–320)	855 (835–875)	335 (320–355)
IP/IV ratio	1.2	1.1	1.3

Our data show that deviation between IP and IV injection in the median lethal dose (LD_50_) results in a ratio IP/IV equal to 1.2, 1.1 and 1.3 for Am, Bo and Aah venoms, respectively. Consequently, the route of administration has little effect on the scorpion venom toxicity level.

### 2.2. Lethal Potency of Representative Snake Venoms

The same procedure was used for scorpion and snake venoms. The LD_50_ toxicity values of collected snake venoms, as assessed by IP and IV injection routes in 20 g Swiss mice, were summarized in Table 2. The calculated ratios of LD_50_ values obtained via IP and IV injections in mice are shown. Of note, Cc venom and Ml venoms. For the three snake venoms, the ratios of LD_50_ values obtained by IP and IV injections are approximately equal to 3. These data clearly point out the impact of the injection route on the venom-induced lethal effects (Table 2).

**Table 2:** Median lethal doses (LD_50_) of Cc, Ml, Ba and Nh snake venoms, as determined using IV and IP injection routes (95% confidence intervals and calculated by non-linear regression).

	LD_50_			
	Cc Venom	Ml Venom	Ba Venom	Nh Venom
IV (µg/mouse)	4.9 (4.2–5.7)	8.03 (7.4–8.5)	5.7 (5.1–6.4)	3.3 (2.5–3.8)
IV (µg/kg)	245 (210–285)	402 (370–425)	285 (255–320)	165 (125–190)
IP (µg/mouse)	14.7 (12.9–15.2)	24.1 (23.7–24.6)	17.4 (17.1–17.9)	4.1 (3.8–4.5)
IP (µg/kg)	735 (645–760)	1205 (1185–1230)	870 (855–895)	205 (190–225)
IP/IV ratio	3.0	3.0	3.1	1.2

Nh venom has a higher toxic activity as compared to the tested viper venoms (Cc, Ml and Ba).

The IP/IV ratio of LD_50_ values for Nh venom injected by IP and IV routes is equal to 1.2. Hence, our data show less divergence in the LD_50_ values with regard to the injection route in mice. Nh venom is found to be 1.5, 2.4 and 1.7 fold more toxic than Cc, Ml, and Ba venoms, respectively, when injected by IV route.

Here, we demonstrated that the injection route has a significant impact on the LD_50_ value and affects the relative toxicity of viper venoms. This is not obvious in the case of cobra venom (Nh) for which the route of injection (IV *versus* IP) has only little effects on the observed toxic activity.

In general, the LD_50_ values obtained in animals injected intravenously were lower than those obtained by intraperitoneal injection, meaning more lethal potency.

Viper venoms are approximately three-times less toxic when injected IP rather than IV. This could be attributed at least in part to a difference in venom toxicokinetic parameters. The biodistribution of viper venom (and associated components) from the site of injection to its various targets is presumably slow and partial, consistent with the fact that molecules in viper venoms are medium and high molecular weight (20–60 kDa) compounds that could have a longer residence time at the site of inoculation thereby reducing the venom bioavailability.

In the case of cobra venom (Nh), the LD_50_ value determined by IP route is close to the value obtained by IV injection. Interestingly, this result is consistent with the data obtained with scorpion venoms (see Table 1), and indicates the presence of small size molecules (with molecular weights < 15 kDa) in cobra venom. Such small molecules could have a shorter residence time at the site of inoculation, resulting in an instant and “complete” bioavailability in the blood.

### 2.3. Concluding Remarks

In this study, we demonstrate that the nature of the animal venom (scorpion, snake) under testing could impact the LD_50_ values obtained by IV and IP routes. This influence has been clearly evidenced in the case of viper venoms for which LD_50_ values were increased about three times when venom is injected by the IP route.

In the case of scorpion venoms, the ratio between the LD50 values (IP *versus* IV) was found to be in the range of 1.1 to 1.3, implying that the absorption and biodistribution of toxins are almost complete and fast. This phenomenon may rely on low molecular weight toxins (<7 kDa) favoring a fast diffusion of molecules into the bloodstream [3,9,10,11,16].

In the case of viper venoms, we highlighted that the LD_50_ values strongly depend on the injection mode. Indeed, the data indicate a 3-fold lower toxicity in IP as compared to IV injection, suggesting a slow and/or partial distribution of the viper venoms (and related components) from the site of injection to its targets. This fact could be attributed—at least in part—to the size of the molecules in viper venoms, such as the hemorrhagic compounds (MW ranging from 39 to 67 kDa), anti-coagulants (MW from 72 to 74 kDa), oedematous proteases and molecules with caseinolytic and amidolytic activities (MW from 29 to 39 kDa and 75 to 100 kDa) [1,5,7,14,17,18].

In the case of cobra venom (Nh), the LD_50_ values obtained by IV and IP injections are almost similar. The toxicity of the potent *Naja haje* venom is mainly due to low molecular weight (generally <30 kDa) toxins, which are likely to quickly diffuse into the bloodstream [5].

It is worth mentioning that the differential toxic/lethal effects observed when these venoms are injected with distinct routes of administration (IP and IV) can be the result of various phenomena related to the absorption and distribution of venom components in the bloodstream, including the size of lethal molecules [19,20,21,22].

## 3. Experimental Section

### 3.1. Snake Venoms

Snake venoms of *Cerastes cerastes*, *Macrovipera lebetina mauretanica*, *Bitis arietans* and *Naja haje* species were extracted by manual stimulation, centrifuged, lyophilized and kept frozen at a temperature of −20 °C at the experimental center of the Pasteur Institute of Morocco, until used [5].

### 3.2. Scorpion Venoms

Am, Aah and Bot scorpion venoms were collected by the electrical stimulation method [23]. Venom was recovered using distilled water and centrifuged (10,000 *g*). The supernatant was lyophilized (freeze dried), and then kept at a temperature of −20 °C prior to use.

### 3.3. Measurement of Protein Concentration

Protein concentrations were accurately determined by the standard method of UV absorbance measurements at a wavelength of 280 nm, with an extinction coefficient ε_1%_ (280 nm) of 10 [24]. For each venom, a venom solution was prepared at a final protein concentration of 5 mg/mL (as determined by A_280 nm_).

### 3.4. Determination of the Median Lethal (LD_50_) Dose

Lethal potency (LD_50_) of venoms (in micrograms of dry weight per mouse) was determined as recommended by the World Health Organization [4,13]. Groups of six mice were used per venom dose; venom concentration was diluted in 150 mM NaCl and injected in final volume of 500 µL by intravenous and intraperitoneal routes.

Mortality was recorded 24 h after injection. The median lethal dose was determined by the method of Software package Prism 5 GraphPad, Inc. (San Diego, CA, USA) according to the provided algorithm. Briefly a non-linear curve fitting (variable slope) was generated using the four-parameter logistical equation; constraints were imposed on minimum (0% mortality) and maximum (100% mortality) values, and no weighing was used. The same package was used to calculate median doses (Anova). Plots were generated using KaleidaGraph 4.03 (Synergy Software, Reading, PA, USA).

### 3.5. Ethics Committee Approval

All the testing and procedures involving animals strictly followed the ethical principles in animal research adopted by the World Health Organization [13]. They were approved by a local ethics committee.

## 4. Conclusions

This study aims at evaluating the lethal potency of a series of scorpion and snake venoms, injected in mice either by the intraperitoneal or intravenous route. Our work should be extended to a greater number of scorpion/snake venoms for a more complete analysis. Also, other representative routes of venom injection (*i.e.*, intracerebroventricular, intramuscular, and subcutaneous) might be examined *in vivo* to assess the actual toxic potential of the various venoms. Obviously, it would be of utmost interest to further evaluate the toxic properties of venoms from other animal species, such as spiders, sea anemones, cone snails, insects, and worms. However, an access to sufficient amounts of natural venoms remains challenging.
